# Nitric Oxide Signal, Nitrogen Metabolism, and Water Balance Affected by γ-Aminobutyric Acid (GABA) in Relation to Enhanced Tolerance to Water Stress in Creeping Bentgrass

**DOI:** 10.3390/ijms21207460

**Published:** 2020-10-10

**Authors:** Mingyan Tang, Zhou Li, Ling Luo, Bizhen Cheng, Youzhi Zhang, Weihang Zeng, Yan Peng

**Affiliations:** 1 Department of Grassland Science, College of Animal Science and Technology, Sichuan Agricultural University, Chengdu 611130, China; tang1976621150@163.com (M.T.); 18581531061@163.com (L.L.); Chengbizhengrass@163.com (B.C.); lotus106601@126.com (Y.Z.); zengwh0123@163.com (W.Z.); pengyanlee@163.com (Y.P.); 2 Institute of Turfgrass Science, Beijing Forestry University, Beijing 100083, China

**Keywords:** *Agrostis Stolonifera*, amino acid metabolism, GABA, metabolic homeostasis, nitrogen mobilization, osmotic adjustment, oxidative damage, signal transduction

## Abstract

γ-Aminobutyric acid (GABA) plays an important role in regulating stress tolerance in plants. Purposes of this study was to determine the effect of an exogenous supply of GABA on tolerance to water stress in creeping bentgrass (*Agrostis stolonifera*), and further reveal the GABA-induced key mechanisms related to water balance, nitrogen (N) metabolism and nitric oxide (NO) production in response to water stress. Plants were pretreated with or without 0.5 mM GABA solution in the roots for 3 days, and then subjected to water stress induced by −0.52 MPa polyethylene glycol 6000 for 12 days. The results showed that water stress caused leaf water deficit, chlorophyll (Chl) loss, oxidative damage (increases in superoxide anion, hydrogen peroxide, malondialdehyde, and protein carbonyl content), N insufficiency, and metabolic disturbance. However, the exogenous addition of GABA significantly increased endogenous GABA content, osmotic adjustment and antioxidant enzyme activities (superoxide dismutase, catalase, dehydroascorbate reductase, glutathione reductase and monodehydroascorbate reductase), followed by effectively alleviating water stress damage, including declines in oxidative damage, photoinhibition, and water and Chl loss. GABA supply not only provided more available N, but also affected N metabolism through activating nitrite reductase and glutamine synthetase activities under water stress. The supply of GABA did not increase glutamate content and glutamate decarboxylase activity, but enhanced glutamate dehydrogenase activity, which might indicate that GABA promoted the conversion and utilization of glutamate for maintaining Chl synthesis and tricarboxylic acid cycle when creeping bentgrass underwent water stress. In addition, GABA-induced NO production, depending on nitrate reductase and NO-associated protein pathways, could be associated with the enhancement of antioxidant defense. Current findings reveal the critical role of GABA in regulating signal transduction and metabolic homeostasis in plants under water-limited condition.

## 1. Introduction

The occurrence of drought is rising with the change of global climate and the decrease in available water resources. Drought stress causes plant growth inhibition and damages normal biological and metabolic processes in plants, such as causing a decline in photosynthetic rate, an increase in oxidative damage, and accelerated leaf senescence [1,2]. Previous studies have shown that plant growth regulators (PGRs) can be used to enhance plant tolerance to water stress [3,4]. γ-Aminobutyric acid (GABA) is a four-carbon non-protein amino acid, and was firstly studied in animals. In recent years, it has been found that GABA plays an important role in regulating growth and is also beneficial to carbon and nitrogen (N) balance, cytoplasmic pH regulation, oxidative balance and osmotic adjustment (OA) under abiotic stress [5,6]. Drought stress can cause a significant increase of GABA content in soybean (*Glycine max*) leaves [7]. Liu et al. showed that the exogenous application of GABA could increase endogenous GABA content and effectively alleviate heat damage in creeping bentgrass (*Agrostis stolonifera*) [8]. Liu et al. found that tobacco (*Nicotiana tabacum*) plants significantly increased GABA biosynthesis in response to water stress, and GABA could act as an effective regulator to enhance antioxidant defense [9]. GABA affected the aluminum-activated malic acid transporter involved in regulating plant growth and stress response [10]. Exogenous GABA, as an important N source, maintained the normal growth of *Arabidopsis thaliana* under low N stress [11]. GABA application improved antioxidant enzyme activities and reduced the accumulation of reactive oxygen species (ROS) and malondialdehyde (MDA), thus maintaining cell membrane stability in rice (*Oryza sativa*) under high temperature stress [12]. These previous studies illustrated the multiple functions of GABA in affecting stress tolerance in plants. However, GABA-regulated water and metabolic homeostasis, associated with tolerance to water stress, are still not fully demonstrated in plants.

The N metabolism is one of the most basic physiological processes in plants due to the primary roles of N as a nutrient and in important components of many compounds, such as proteins, nucleic acids, enzymes, chlorophyll, vitamins, plant hormones and alkaloids [13]. The N metabolism affects plant growth and development, quality and yield of crops, and resistance to environmental stress [14]. Under drought stress, a higher N content could improve photosynthesis, antioxidant enzyme activities, and proline accumulation associated with better drought tolerance in rice [15]. N is also well known for its effects on reducing leaf senescence and enhancing heat tolerance under high temperature conditions [16,17]. Nitric oxide (NO) is an important product of N metabolism. As an important signaling molecule, NO plays a positive role in regulating various physiological processes in plants under normal and adverse conditions [18]. NO-associated protein (NOA) and nitrate reductase (NR) pathways dependent on N metabolism are the two main pathways of NO production in plants [19]. Under drought stress, NO promoted the wheat (*Triticum aestivum*) seed germination rate, embryo length, endopeptidase and amylase activities, and also increased antioxidant enzyme activities and proline content in wheat [20]. Under salt and heat stress, a low concentration of NO pretreatment could enhance photosystem II quantum production and antioxidant enzyme activities, thus improving the survival rate of rice seedlings [21]. NO treatment could alleviate the drought damage in wheat seedlings [22] and also effectively reduced the growth inhibition and lipid peroxidation in *Elymus nutans* under cold stress [23]. Until now, the role of GABA in regulating NO production, contributing to enhanced tolerance to water stress in plant species, has been unclear.

Creeping bentgrass is an excellent gramineous turfgrass and is widely used on sports lawns, such as golf course, bowling lawns and tennis courts, due to its fine texture and resistance to low and frequent mowing. However, creeping bentgrass is a cold-season shallow-rooted turfgrass with lower drought tolerance [24]. The purpose of this study was to determine whether the exogenous application of GABA could significantly improve tolerance to water stress based on phenotypic and physiological changes, and further reveal the GABA-induced key mechanism related to antioxidant defense, water balance, N metabolism and NO production in creeping bentgrass under water stress.

## 2. Results

### 2.1. Cell Membrane Stability, Water Status, and Photosynthesis Affected by GABA

**Figure 1:**
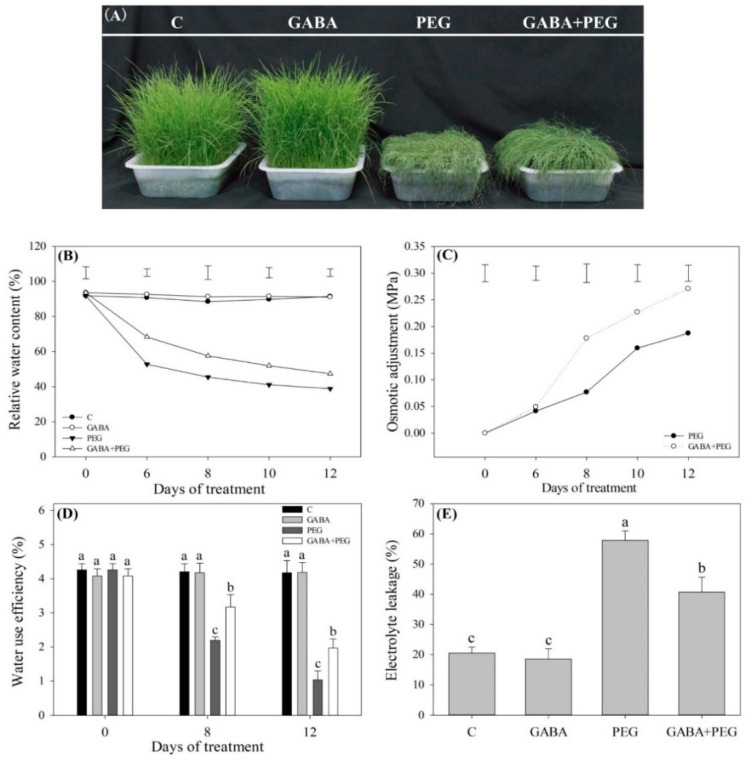
Effects of γ-aminobutyric acid (GABA) pretreatment on (**A**) phenotype, (**B**) relative water content (RWC), (**C**) osmotic adjustment (OA), (**D**) water use efficiency (WUE) and (**E**) electrolyte leakage (EL) in leaves of creeping bentgrass under non-stress and water stress conditions. Vertical bars above curves in (**A**) or (**B**) represent the least significant difference (LSD) values at a particular day (*n* = 4; *P* ≤ 0.05). Vertical bars above the columns in (**C**) or (**D**) indicate ± SE of mean (*n* = 4) and different letters above columns indicate significant difference at a particular day based on the LSD (*P* ≤ 0.05). C, well-watered control; GABA, well-watered control pretreated with GABA; PEG, PEG-induced water stress; GABA + PEG, water-stressed plants pretreated with GABA.

**Figure 2:**
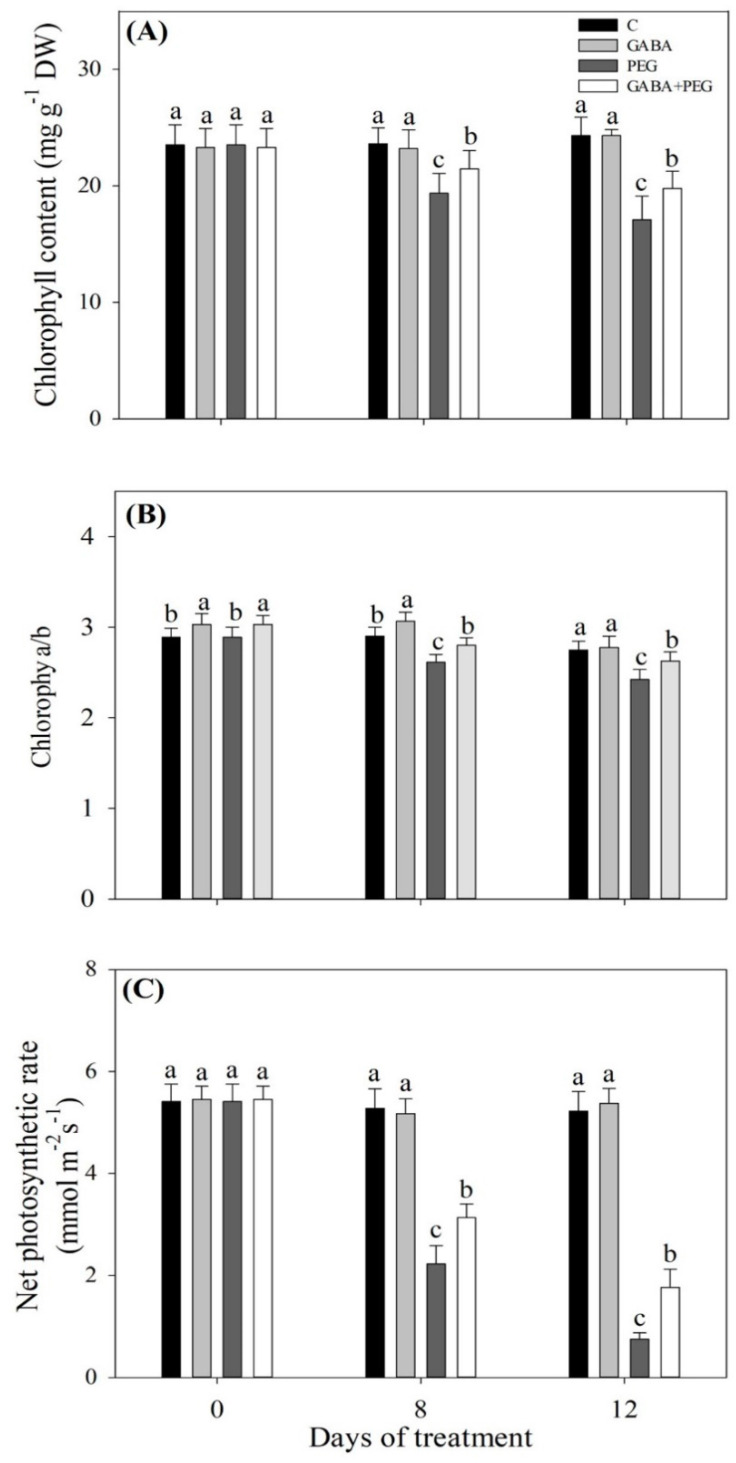
Effects of γ-aminobutyric acid (GABA) pretreatment on (**A**) total chlorophyll content, (**B**) chlorophyll a/b, and (**C**) net photosynthetic rate in leaves of creeping bentgrass under non-stress and water stress conditions. Vertical bars indicate ± SE of mean (*n* = 4). Different letters above columns indicate significant difference at a particular day based on the LSD (*P* ≤ 0.05). C, well-watered control; GABA, well-watered control pretreated with GABA; PEG, PEG-induced water stress; GABA + PEG, water-stressed plants pretreated with GABA.

Phenotypic changes showed that water stress resulted in leaves wilting (Figure 1A). Leaf relative water content (RWC) significantly decreased, but the exogenous supply of GABA could significantly alleviate stress-induced decline in RWC during 12 days of water stress (Figure 1B). Osmotic adjustment (OA) gradually increased with the prolonged period of water stress, and GABA-treated plants exhibited significantly higher OA than untreated plants at 8, 10 and 12 days of water stress (Figure 1C). Water stress decreased water use efficiency (WUE) in leaves, whereas GABA-treated plants could maintain significantly higher WUE than untreated plants at 8 and 12 days of water stress (Figure 1D). Electrolyte leakage (EL) obviously increased when GABA-treated and untreated plants responded to water stress, but GABA-treated plants exhibited a 29.6% decrease in EL as compared to untreated plants under water stress (Figure 1E). When plants were subjected to water stress, total chlorophyll (Chl), Chla/b, and net photosynthetic rate (Pn) decreased significantly (Figure 2A–C). GABA-treated creeping bentgrass maintained significantly high levels of Chl, Chla/b and Pn under water stress (Figure 2A–C).

### 2.2. Oxidative Damage and Antioxidant Enzyme Activities Affected by GABA

**Figure 3:**
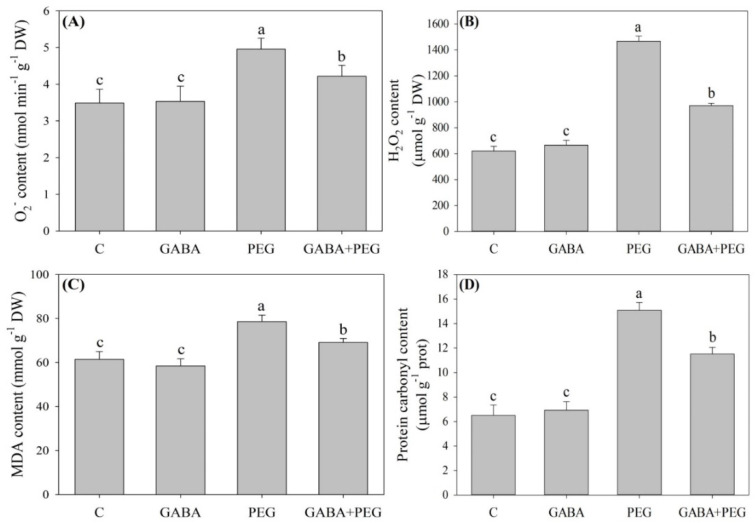
Effects of exogenous γ-aminobutyric acid (GABA) pretreatment on (**A**) superoxide anion (O_2_^−^) content, (**B**) hydrogen peroxide(H_2_O_2_) content, (**C**) malondialdehyde (MDA) content, and (**D**) protein carbonyl content in leaves of creeping bentgrass under non-stress and water stress conditions. Vertical bars indicate ± SE of mean (*n* = 4). Different letters above columns indicate significant difference based on the LSD (*P* ≤ 0.05). C, well-watered control; GABA, well-watered control pretreated with GABA; PEG, PEG-induced water stress; GABA + PEG, water-stressed plants pretreated with GABA.

**Figure 4:**
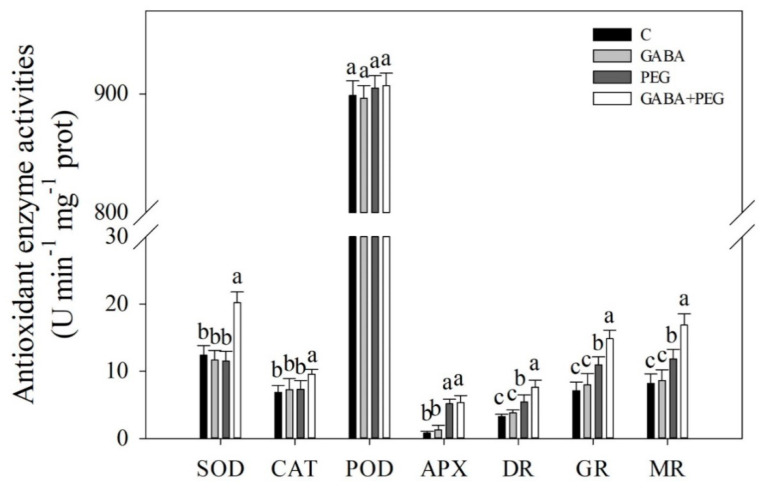
Effects of exogenous γ-aminobutyric acid (GABA) pretreatment on antioxidant enzyme activity in leaves of creeping bentgrass under non-stress and water stress conditions. SOD, superoxide dismutase activity; CAT, catalase activity; POD, guaiacol peroxidase activity; APX, ascorbate peroxidase activity; DR, dehydroascorbate reductase activity; GR, glutathione reductase activity; MR, monodehydroascorbate reductase activity. Vertical bars indicate ± SE of mean (*n* = 4). Different letters above columns indicate significant difference based on the LSD (*P* ≤ 0.05). C, well-watered control; GABA, well-watered control pretreated with GABA; PEG, PEG-induced water stress; GABA + PEG, water-stressed plants pretreated with GABA.

Superoxide anion (O_2_^−^), hydrogen peroxide (H_2_O_2_), MDA, and protein carbonyl content were not affected significantly by GABA pretreatment under normal conditions (Figure 3A–D). Water stress induced significant increases in O_2_^−^, H_2_O_2_, MDA and protein carbonyl content in leaves, and exogenously applied GABA reduced the accumulation of O_2_^−^, H_2_O_2_, MDA and protein carbonyl content in the leaves of creeping bentgrass in response to water stress (Figure 3A–D). GABA-treated plants showed 14.9%, 33.8%, 12% or 23.7% decreases in O_2_^−^, H_2_O_2_, MDA, and carbonyl content compared to untreated plants under water stress, respectively (Figure 3A–D). Water stress led to increases in peroxidase (POD), ascorbate peroxidase (APX), dehydroascorbate reductase (DR), glutathione reductase (GR) and monodehydroascorbate reductase (MR) activities, and exogenous GABA further improved stress-induced increases in DR, GR and MR activities in leaves after 12 days (Figure 4). The superoxide dismutase (SOD) and catalase (CAT) activities were not changed by water stress in plants without GABA application, but significantly increased in GABA-treated plants under water stress. The GABA pretreatment did affect POD activity under normal conditions and water stress (Figure 4).

### 2.3. Nitrogen Metabolism and Nitric Oxide Affected by GABA

**Figure 5:**
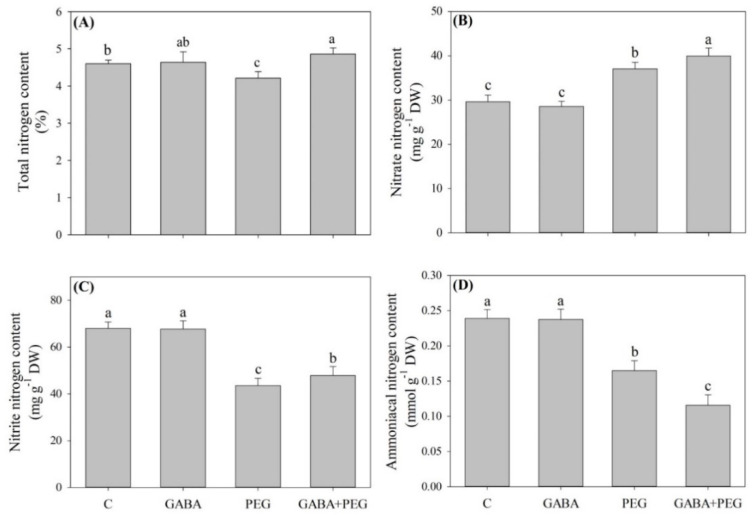
Effects of exogenous γ-aminobutyric acid (GABA) pretreatment on (**A**) total nitrogen content, (**B**) nitrate nitrogen content, (**C**) nitrite nitrogen content and (**D**) ammoniacal nitrogen content in leaves of creeping bentgrass under non-stress and water stress conditions. Vertical bars indicate ± SE of mean (*n* = 4). Different letters above columns indicate significant difference based on the LSD (*P* ≤ 0.05). C, well-watered control; GABA, well-watered control pretreated with GABA; PEG, PEG-induced water stress; GABA + PEG, water-stressed plants pretreated with GABA.

**Figure 6:**
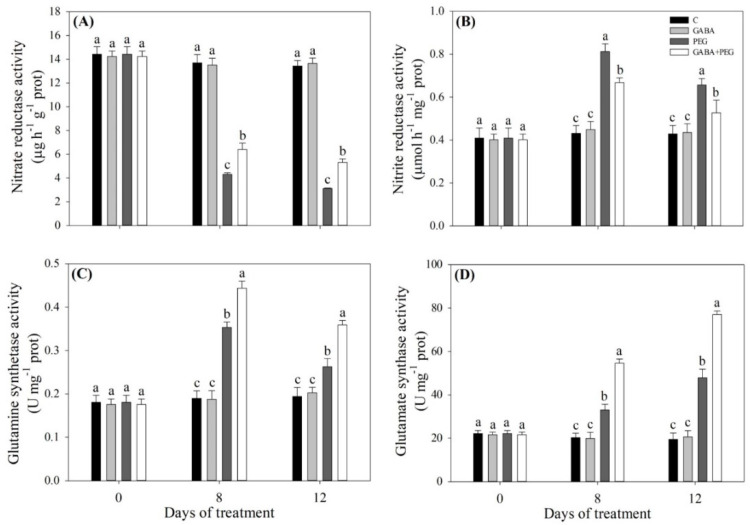
Effects of exogenous γ-aminobutyric acid (GABA) pretreatment on (**A**) nitrate reductase activity, (**B**) nitrite reductase activity, (**C**) glutamine synthetase activity, and (**D**) glutamate synthase activity in leaves of creeping bentgrass under non-stress and water stress conditions. Vertical bars indicate ± SE of mean (*n* = 4). Different letters above columns indicate significant difference at a particular day based on the LSD (*P* ≤ 0.05). C, well-watered control; GABA, well-watered control pretreated with GABA; PEG, PEG-induced water stress; GABA + PEG, water-stressed plants pretreated with GABA.

**Figure 7:**
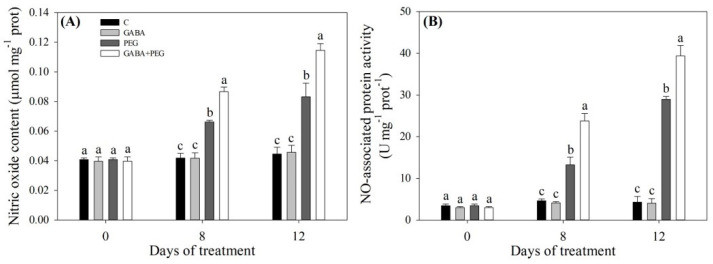
Effects of exogenous γ-aminobutyric acid (GABA) pretreatment on (**A**) nitric oxide content, (**B**) NO-associated protein activity in leaves of creeping bentgrass under non-stress and water stress conditions. Vertical bars indicate ± SE of mean (*n* = 4). Different letters above columns indicate significant difference at a particular day based on the LSD (*P* ≤ 0.05). C, well-watered control; GABA, well-watered control pretreated with GABA; PEG, PEG-induced water stress; GABA + PEG, water-stressed plants pretreated with GABA.

As shown in Figure 5, water stress led to a decrease in total N content in plants without GABA application, but water-stressed plants with GABA pretreatment maintained total N content at a normal level in the leaves (Figure 5A). Under non-stress conditions, exogenous GABA had no significant effects on nitrate N, ammoniacal N or nitrite N content (Figure 5B–D). Water stress promoted the nitrate N accumulation in all treatments, and water-stressed plants without or with GABA application had a 25.23% or 34.93% increase in nitrate N content compared to control, respectively (Figure 5B). Water stress reduced ammonia N and nitrite N content in all treatments (Figure 5C,D). As compared with the control treatment, there was a 27.99% or 49.51% decrease in ammonia N content with the PEG or GABA + PEG treatment, respectively (Figure 5C). GABA-treated plants maintained significantly higher nitrite N than untreated plants under water stress (Figure 5D). The NR activity in leaves decreased sharply under water stress, and GABA-pretreated plants had significantly higher NR activities than untreated plants at 8 and 12 days of water stress (Figure 6A). Nitrite reductase (NiR), glutamine synthetase (GS) and glutamate synthase (GOGAT) activities significantly increased when creeping bentgrass responded to water stress (Figure 6B–D). Exogenous GABA significantly reduced NR activity under water stress (Figure 6B). The GS activity in GABA-treated plants showed a 25.6% or 36.6% increase compared to that in untreated plants at 8 or 12 days of water stress, respectively (Figure 6C). Exogenous GABA also further improved the stress-induced increase in GOGAT activity at 8 and 12 days (Figure 6D). The NO content and NOA activity gradually increased during water stress (Figure 7A,B). The NO content in the leaves of GABA-pretreated plants increased by 30.8% or 37.7% from that of untreated plants at 8 or 12 days of water stress, respectively (Figure 7A). The NOA activity in the leaves of GABA-treated plants was 0.8 or 0.36 times higher than that of untreated plants at 8 or 12 days of water stress, respectively (Figure 7B).

### 2.4. Endogenous Glutamic Acid and GABA Affected by GABA

**Figure 8:**
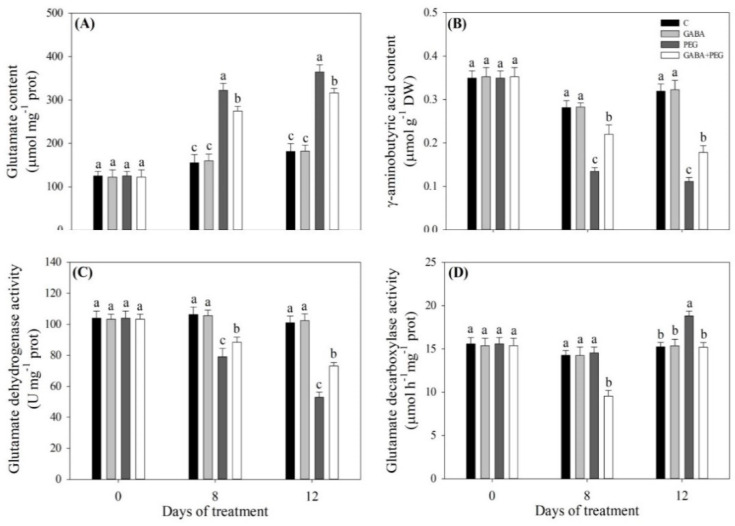
Effects of exogenous γ-aminobutyric acid (GABA) pretreatment on (**A**) glutamate content, (**B**) γ-aminobutyric acid content, (**C**) glutamate dehydrogenase activity, and (**D**) glutamate decarboxylase activity in the leaves of creeping bentgrass under non-stress and water stress conditions. Vertical bars indicate ± SE of mean (*n* = 4). Different letters above columns indicate significant difference at a particular day based on the LSD (*P* ≤ 0.05). C, well-watered control; GABA, well-watered control pretreated with GABA; PEG, PEG-induced water stress; GABA + PEG, water-stressed plants pretreated with GABA.

**Figure 9:**
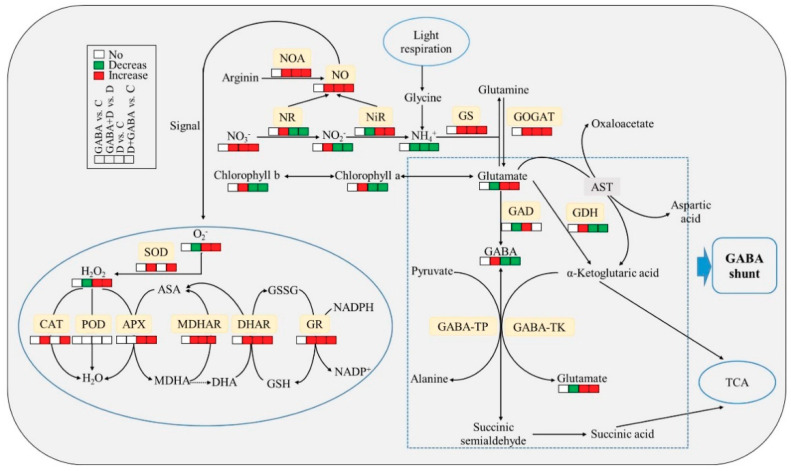
Integrative pathways induced by water stress and exogenous application of γ-aminobutyric acid (GABA) in leaves of creeping bentgrass under non-stress and water stress conditions. Red indicates a significant upregulation, green indicates a significant downregulation, and white indicates no significant change. C, well-watered control; GABA, well-watered control pretreated with GABA; PEG, PEG-induced water stress; GABA + PEG, water-stressed plants pretreated with GABA.

Under normal conditions, the GABA pretreatment had no significant effects on glutamate content, GABA content, glutamate dehydrogenase (GDH) activity or glutamate decarboxylase (GAD) activity in leaves (Figure 8A–D). Water stress significantly improved glutamate (Glu) content, but decreased GABA content in the leaves (Figure 8A,B). Exogenous GABA significantly reduced glutamic acid accumulation, but promoted GABA accumulation under water stress (Figure 8A,B). The GDH activity in the leaf was significantly reduced under water stress, and GABA-treated plants had higher GDH activity than untreated plants at 8 and 12 days of water stress (Figure 8C). There were no significant differences in GAD activity among four treatments at 0 day, and the GABA + PEG treatment exhibited significantly lower GAD activity than the other three treatments at 8 days (Figure 8D). Water stress improved GAD activity, but exogenous GABA application reduced the stress-induced increase in GAD activity at 12 days (Figure 8D). Figure 9 shows the integrative pathways induced by water stress and exogenous GABA application in the leaves of creeping bentgrass.

## 3. Discussion

Drought stress leads to an imbalance in water, stomatal closure, destruction of membrane integrity, and disorder of other physiological processes in plant cells [25,26,27,28]. Drought-caused stomatal closure reduces transpiration rates and gaseous exchange, which seriously inhibits the photosynthetic rate of plants and plant growth [29,30]. A previous study found that foliar application of GABA could significantly alleviate leaf wilting and Chl loss, and also improved OA and photosynthesis in the leaves of creeping bentgrass under heat stress [31]. In the current study, creeping bentgrass pretreated with GABA in the roots significantly alleviated stress-induced leaf water loss and led to a decline in Pn, which could be due to the enhanced OA and lower Chl loss in leaves under water stress. In addition to changes of water status and photosynthesis, enhanced ROS production, including O_2_^−^ and H_2_O_2_, is one of inevitable consequences of water stress in different cellular compartments [32]. Antioxidant enzymes, such as SOD, CAT, POD and enzymes involved in the ascorbic acid-glutathione (ASA-GSH) cycle (APX, DR, GR, and MR), are key components of the antioxidant defense that removes O_2_^−^, H_2_O_2_ and other free radicals, thereby maintaining a proper redox balance in cells under stress conditions [33]. Protein carbonyl is one of products of protein peroxidation is and widely used as a marker of oxidative damage in plants [34]. It has been found that foliar GABA pretreatment significantly reduced O_2_^−^, H_2_O_2_ and MDA content through upregulating transcript levels of genes encoding POD, APX and GR in the leaves of creeping bentgrass under heat and drought stress [35]. This is similar to the present findings: the application of GABA increased SOD, CAT, DR, GR and MR activities associated with significant decreases in O_2_^−^, H_2_O_2,_ MDA, and protein carbonyl content in creeping bentgrass under water stress. As stated above, GABA-pretreated creeping bentgrass showed less stress damage than untreated plants in response to water stress, which could be related to a GABA-induced improvement in OA and antioxidant defense.

Previous research revealed that GABA can be used as a temporary N pool and source in plants [36]. The N content decreased due to the inhibition of the GABA biosynthesis in *Arabidopsis thaliana* mutant under hypoxic condition [37]. However, N metabolism and conversion may be more important than N accumulation when plants suffer from water stress. In the present study, water stress significantly decreased the total N in the leaves of creeping bentgrass, but exogenous GABA supply elevated N content in creeping bentgrass under water stress. This is consistent with previous studies on the function of GABA as an N source in plants. N uptake and conversion is an important metabolic process for plant growth and adaption to stress. NR catalyzes the conversion of NO_3_^−^ into NO_2_^−^, which is reduced by NiR to generate NH_4_^+^. NH_4_^+^ can be assimilated into organic N for maintaining N balance [38]. NiR activity increased firstly and then decreased in apple (*Malus domestica*) leaves under drought stress [39]. Exogenous GABA maintained N metabolism balance in muskmelon (*Cucumis melo*) seedlings under Ca (NO_3_)_2_ stress [40]. Our results found that drought stress enhanced NO_3_^−^ accumulation, but decreased the NO_2_^-^ and NH_4_^+^ content in creeping bentgrass. The exogenous addition of GABA promoted the conversion of NO_3_^−^ to NO_2_^−^ by increasing NR activity in the leaves of creeping bentgrass under water stress. These findings suggest that GABA supply not only provided more N, but also affected N metabolism when creeping bengtgrass responded to water stress.

GS in plants is involved in NH_4_^+^ assimilation, which is an important pathway for the conversion of inorganic N to organic glutamine. This metabolic process also reduces ammonia’s toxicity to plants under adverse environmental conditions. In plants, GOGAT catalyzes the conversion of glutamine into glutamate, which is a key intermediate amino acid for synthesizing other amino acids, such as GABA by GAD and glutamine by GS. In addition, GDH catalyzes the conversion of glutamate to α-ketoglutaric acid, which is an intermediate of the tricarboxylic acid cycle (TCA) cycle [41,42,43]. In our study, the exogenous addition of GABA could effectively alleviate the water stress-caused decline in GABA in creeping bentgrass. GABA application enhanced NH_4_^+^ assimilation, and also improved the interchange between glutamate and glutamine through activating GS and GOGAT in the leaves of creeping bentgrass under water stress. Interestingly, exogenous GABA did not increase glutamate content and GAD activity, but enhanced GDH activity in creeping bentgrass. These findings might indicate that GABA promoted the conversion and utilization of glutamate for maintaining Chl synthesis and the TCA cycle when creeping bentgrass was exposed to water stress. A previous analysis of metabolomics found that the foliar application of GABA could increase the accumulation of intermediate metabolites of the TCA cycle, implying the function of GABA in maintaining metabolic homeostasis in creeping bentgrass under heat and drought conditions [31,44]. However, GABA-regulated N and the amino acid metabolism associated with drought tolerance in perennial plants still deserve to be studied in the future.

There are three main pathways for NO synthesis, namely the NOA pathway, the NR pathway, and the NiR pathway in plants [45,46,47,48]. As a very important second signal molecule, NO regulates the growth and development of plants and stress tolerance under biotic and abiotic stresses [49]. Many studies have reported that the NO signal helps plants to survive stressful conditions through activating antioxidative enzymes to alleviate oxidative damage. For example, NO could improve the drought tolerance of white clover (*Trifolium repens*) associated with increases in SOD, CAT, APX, and POD activity, as well as gene expression [50]. NO treatment effectively alleviated drought-induced oxidative damage in relation to increases in SOD and POD activities in wheat seedlings [22]. The drought tolerance of *Dendrobium huoshanense* could be improved by exogenous NO through promoting different antioxidant enzyme (SOD, CAT and POD) activities, photosynthetic capacity, and Chl content under drought stress [51]. The results of the current study show that the exogenous addition of GABA significantly increases NO content, which could be associated with the activation of SOD, CAT, DR, GR and MR activities in the leaves of creeping bentgrass under water stress. The study of Peng et al. [50] found that an enhanced antioxidant defense was regulated by the spermidine-induced NO signal, depending on the NOA and NR pathways, when white clover suffered from water stress, which was consistent with our present findings in creeping bentgrass.

## 4. Materials and Methods

### 4.1. Plant Materials and Treatments

Seeds of creeping bentgrass cv. ‘Penncross’ were purchased from the Tee-2-Green company, Oregon, USA. All seeds were quickly sterilized with 0.1% mercuric chloride solution for 3 min, and then rinsed 3 times with distilled water. Seeds (0.39 g) were germinated in a plastic container (24 cm length, 18 cm width, 9 cm depth) with sterile quartz sand in a controlled growth chamber (14 h photoperiod cycle, 23/19 °C day/night temperature, 65% relative humidity, and 750 μmol photon m^−2^ s^−^^1^ photosynthetic active radiation). After 7 days of germination, the seedlings were irrigated with Hoagland’s solution [52] for 23 days. For the 3 days of GABA pretreatment, seedlings were cultivated in the Hoagland’s solution containing 0.5 mmol/L GABA [53], and the GABA-untreated seedlings were cultivated in the normal Hoagland’s solution for 3 days. The 33-day-old GABA-pretreated and untreated seedlings were then subjected to normal cultivation or water stress induced by polyethylene glycol (PEG) 6000 (−0.52 MPa) for 12 days. The PEG 6000 was dissolved in the Hoagland’s solution. The Hoagland’s and PEG solutions were refreshed every day. All plants were arranged in a completely randomized design. Each treatment had four independent biological replicates (four containers) and a container was regarded as a biological replicate. Leaves were sampled at 0, 6, 8, 10 and 12 days under well-watered and water stress conditions.

### 4.2. Measurements of Cell Membrane Stability, Water Status and Photosynthesis

For relative EL, 0.1 g of fresh leaves were fully soaked in distilled water for 24 h and the initial conductivity (S_1_) of the solution was detected using a conductivity meter (model 32; Yellow Springs Instrument Co., Yellow Spring, OH). After being autoclaved for 15 min at 100 °C, the final conductivity (S_2_) of the solution was detected. EL was calculated according to EL (%) = S_1_/S_2_ × 100% [54]. For, leaf RWC, the method of [55] was used and 0.2 g of fresh leaves were collected for the determination of RWC. Osmotic potential (OP) in the leaf was detected by using the method of [56]. Leaves (0.2 g) were collected and immediately frozen in liquid N for 10 min and thawed at 4 °C for 25 min. The cell liquid was put into the sampling chamber of an osmometer (Wescor, Logan, UT, USA) to get the osmolality. OP was calculated based on MPa = −c × 2.58 × 10^−3^. The OP of control leaves was subtracted from the OP of stressed leaves to get the OA in leaves. For Chl content in leaves, 0.1 g of leaves was submerged in 10 mL of 95% ethanol and 85% acetone (1:1 *v:v*) for 48 h in the dark and the absorbance of the extraction was measured at 663 nm and 645 nm [57]. Pn and instantaneous WUE were estimated using a portable photosynthetic system (CIRAS-3, PP Systems, USA).

### 4.3. Determination of Oxidative Damage and Antioxidant Enzyme Activities

H_2_O_2_ or O_2_^−^ contents were determined according to the method of [58] or [59], respectively. The carbonyl content of protein was determined by using Test Kits of Suzhou Comin biotechnology company, China. The 0.15 g leaf samples were used to detect H_2_O_2_, O_2_^−^, or carbonyl content for a replicate of each treatment. For MDA content, soluble protein content and antioxidant enzyme activities, leaves (0.1 g) were ground with 1.5 mL of pre-cooled phosphate buffer (150 mM, pH 7.0) and centrifuged at 15,000 g for 20 min at 4 °C. The supernatant was collected for determining MDA content [60], soluble protein content [61], SOD activity [62], POD and CAT activities [63], APX activity [64], and GR, MR and DR activity [65], respectively. For POD, CAT, APX, DHAR, GR and MDHAR activity, the absorbance of the reaction solution was monitored every 10 s for 1 min.

### 4.4. Determination of Nitrogen and γ-Aminobutyric Acid Metabolism

Total N content was determined via the kjeldahl method [66]. The samples were crushed, weighed, and packed with 0.2 g of filter paper, and then put into the de-boiling tube. One de-boiling piece was added to each tube, and 5 mL of sulfuric acid was added. There was a blank filter paper control in each batch of de-boiling tube. For nitrate N, the 2 g of dry leaf samples was added into 10 mL of deionized water, and then were heated in a boiling water bath for 30 min. After filtering, 0.1 mL of filtrate was mixed with 0.4 mL of 0.5% salicylic acid–sulfuric acid solution. After homogenization, 9.5 mL of 8% sodium hydroxide solution was slowly added into the supernatant and the absorbance of the mixture was measured at 450 nm [67]. For nitrite N, 0.2 g dry sample powder was added into 70 mL water and 12 mL 20 gL^−1^ sodium hydroxide solution. The 10 mL of zinc sulfate solution was added and then heated in a 60 °C water bath for 10 min. After filtering with filter paper, 1 mL of filtrate was added into 4.5 mL of ammonium chloride buffer and then 2.5 mL of 60% acetic acid, 2.5 mL of 1 gL^−1^ N-1-naphthylethylenediamine solution and 2.5 mL of 10gL^−1^ p-aminobenzene sulfonic acid were added in turn. The mixture was placed for 25 min at room temperature and the absorbance was measured at 550 nm [68]. For ammonium N, 1 g of dry sample powder was mixed with 10 mL of deionized water for 12 h. After filtering, 4 mL of filtrate was added into 1 mL of TCA and the mixture was held at 4 °C for 12 h. After being centrifuged at 10,000× *g* for 5 min, 50 μL of the supernatant was mixed with 50 μL of distilled water, 2.5 mL of phenol reagent, and 2 mL of sodium hypochlorite, and then the mixture was heated in a 95 °C water bath for 5 min. The absorbance of the mixture was detected at 630 nm [69].

For GOGAT and GDH activities, 0.15 g of leaf tissues was ground with 2 mL of 10 mM Tris-HCl buffer (pH 7.6) containing 1 mM MgCl_2_, 1 mM EDTA and 1 mM 2-mercaptoethanol, and the homogenate was centrifuged at 12,000× *g* for 30 min. The supernatant was used for analyzing enzyme activities. For GOGAT activity, the reaction mixture contained 0.4 mL of 20 mM L-glutamine, 0.05 mL of 0.1 M α-ketoglutaric acid, 0.1 mL of 10 mM potassium chloride, 0.2 mL of 3 mM NADH, 0.5 mL of the supernatant, and 1.75 mL of 25 mM Tris-HCL buffer (pH 7.6). The reaction started with the addition of L-glutamine and the absorbance was recorded immediately at 340 nm (OD_1_), and then the absorbance was recorded again at 340 nm after 3 min (OD_2_) [70]. For GDH activity, the reaction mixture contained 0.3 mL of 0.1 M α-ketoglutaric acid, 0.3 mL of 1 M NH_4_Cl, 0.2 mL of 3 M NADH, 1 mL of the supernatant, and 1.2 mL of 0.2 M Tris-HCL buffer (pH 8.0). The absorbance of the reaction solution was recorded immediately at 340 nm (OD_1_), and then the absorbance was recorded again at 340 nm after 3 min (OD_2_) [71]. The Glu content (Art. No. GLU-2-Y), NR activity (Art. No. NR-2-Y), NiR activity (Art. No. NIR-2-G) and NO content (Art. No. NO-2-G) were measured using Assay Kits from Suzhou Comin Biotechnology Co., Ltd., China. GABA content (Art. No. ml027467) was measured using a kit sold by mlbio Good elisakit producers, Shanghai, China. The GS activity (Art. No. G0401F) and GAD activity (Art. No. G1102F) were measured using a kit sold by Suzhou Grace Biotechnology Co., Ltd., China. The NOA activity (Art. No. A014-2-2) was measured using the Assay Kit (NanJing Jian Cheng Bioengineering Institute., China). For the determination of these parameters, 0.2 g leaf samples were used for a replicate of each treatment.

### 4.5. Statistical Analysis

All data was analyzed by using SPSS 20 (IBM, Armonk, NY, USA) and the SAS (SAS 9.1, SAS Institute, Cary, NC). Differences among treatments were determined by using the Fisher’s protected least significance difference (LSD) test at *P* ≤ 0.05. The two-way ANOVA was made before using Fisher’s LSD.

## 5. Conclusions

Water stress caused leaf water deficit, Chl loss, oxidative damage, N insufficiency and metabolic disturbances in creeping bentgrass. However, the exogenous addition of GABA effectively alleviated water stress damage, including reductions in oxidative damage, photoinhibition, and water and Chl loss, which could be related to the GABA-induced improvement of OA and antioxidant defense. GABA supply not only provided more available N, but also affected N metabolism through activating NR and GS activities when creeping bentgrass was exposed to water stress. The exogenous addition of GABA did not increase glutamate content and GAD activity, but enhanced GDH activity, which might indicate that GABA promoted the conversion and utilization of glutamate for maintaining Chl synthesis and the TCA cycle in creeping bentgrass under water stress. In addition, GABA-induced NO production, dependent on the NOA and NR pathways, could be associated with the enhancement of antioxidant defenses in creeping bentgrass. Current findings further reveal the critical role of GABA in regulating signal transduction and metabolic homeostasis in plants under water stress.

## Abbreviations

GABA	γ-aminobutyric acid
PEG-6000	polyethylene glycol-6000
SOD	superoxide dismutase
CAT	catalase
POD	peroxidase
APX	ascorbate peroxidase
DR	dehydroascorbate reductase
GR	glutathione reductase
MR	monodehydroascorbate reductase
RWC	relative water content
WUE	water use efficiency
Pn net	photosynthetic rate
EL	electrolyte leakage
OP	osmotic potential
OA	osmotic adjustment
Chl	total chlorophyll
TCA	tricarboxylic acid
NO	nitric oxide
NOA	NO-associated protein
NR	nitrate reductase
NiR	nitrite reductase
Glu	glutamate
GS	glutamine synthetase
GOGAT	glutamate synthase
GDH	glutamate dehydrogenase
GAD	glutamate decarboxylase
PGRs	plant growth regulators
ROS	reactive oxygen species
MDA	malondialdehyde
O_2_^−^	superoxide anion
H_2_O_2_	hydrogen peroxide
